# The Local Intraarterial Administration of Nimodipine Might Positively Affect Clinical Outcome in Patients with Aneurysmal Subarachnoid Hemorrhage and Delayed Cerebral Ischemia

**DOI:** 10.3390/jcm11072036

**Published:** 2022-04-05

**Authors:** Johannes Walter, Martin Grutza, Markus Möhlenbruch, Dominik Vollherbst, Lidia Vogt, Andreas Unterberg, Klaus Zweckberger

**Affiliations:** 1 Department of Neurosurgery, University of Heidelberg, Im Neuenheimer Feld 400, 69120 Heidelberg, Germany; martin.grutza@med.uni-heidelberg.de (M.G.); lidia.vogt@med.uni-heidelberg.de (L.V.); andreas.unterberg@med.uni-heidelberg.de (A.U.); k.zweckberger@klinikum-braunschweig.de (K.Z.); 2 Department of Neuroradiology, University of Heidelberg, Im Neuenheimer Feld 400, 69120 Heidelberg, Germany; markus.moehlenbruch@med.uni-heidelberg.de (M.M.); dominik.vollherbst@med.uni-heidelberg.de (D.V.)

**Keywords:** subarachnoid hemorrhage, aneurysmal subarachnoid hemorrhage, hemorrhagic stroke, rescue therapy, aneurysm, neuropsychology

## Abstract

The effect of the intraarterial administration of nimodipine as a rescue measure to treat delayed vasospasm after aSAH remains understudied; therefore, we evaluated its effect on short- and long-term functional and neuropsychological outcomes after aSAH. In this prospective observational study, a total of 107 consecutive patients treated for aSAH of WFNS grades I–V were recruited. At follow-up visits 3-, 12- and 24-months after the hemorrhage, functional outcome was assessed using the Extended Glasgow Outcome (GOSE) and modified Rankin (mRS) scales, while neurocognitive function was evaluated using the screening module of the Neuropsychological Assessment Battery (NAB-S). The outcome of patients, who had received rescue therapy according to the local standard treatment protocol (interventional group, *n* = 37), and those, who had been treated conservatively (conservative group, *n* = 70), were compared. Even though significantly more patients in the interventional treatment group suffered from high-grade aSAH (WFNS Grades IV and V, 54.1% vs. 31.4%, *p* = 0.04) and required continuous drainage of cerebrospinal fluid at discharge (67.7% vs. 37.7%, *p* = 0.02) compared to the control group, significant differences in functional outcome were present only at discharge and three months after the bleeding (GOSE > 4 in 8.1% vs. 41.4% and 28.6% vs. 72.7%, *p* < 0.001 and *p* = 0.01 for the interventional and control group, respectively). Thereafter, group differences were no longer significant. While significantly more patients in the intervention group had severe neuropsychological deficits (76.3% vs. 36.0% and 66.7% vs. 29.2%, *p* = 0.04 and 0.05, respectively) and were unable to work (5.9% vs. 38.1%, *p* = 0.03 at twelve months) at three and twelve months after the hemorrhage, no significant differences between the two groups could be detected at long-term follow-up. The presence of moderate neuropsychological impairments did not significantly differ between the groups at any timepoint. In conclusion, despite initially being significantly more impaired, patients treated with intraarterial administration of nimodipine reached the same functional and neuropsychological outcomes at medium- and long-term follow-up as conservatively treated patients suggesting a potential beneficial effect of intraarterial nimodipine treatment for delayed vasospasm after aSAH.

## 1. Introduction

Aneurysmal subarachnoid hemorrhage (aSAH) can be a devastating and life-changing event, as it leads to significant morbidity and mortality. Aside from primary damage to the brain at the time of bleeding caused by, e.g., intracerebral hemorrhage or transient ischemia, secondary insults, such as the development of delayed cerebral ischemia (DCI), infection or hydrocephalus are the most important predictors of impaired functional outcome in patients suffering from aSAH [1,2]. It has been shown that DCI is the most important secondary injury mechanism associated with increased mortality and poor outcome after aSAH [1,2]. Although vasospasm, microthrombosis, cortical spreading depolarization and failure of cerebral autoregulation have been implicated in the development of DCI, most cases of DCI are the result of vasospasm; therefore, resolving vasospasm has traditionally been one of the main treatment targets of neurointensive care in order to improve the outcome of patients with aSAH [3,4,5]. A plethora of medical treatment options to attenuate vasospasm, such as statins, corticosteroids, clazosentan, milrinone, magnesium sulfate, fasudil hydrochloride, cilostazol and edaravone, has been tested in both, experimental and clinical settings; however, with exemption of one single agent, the calcium channel antagonist nimodipine, no systemic medical therapy has shown a robust and reproducible effect on functional outcome in these patients [6,7,8,9,10,11,12,13,14,15]. Therefore, in most centers, enteral administration of nimodipine is part of the standard treatment protocol in order to prevent vasospasm; however, different routes of administration including parenteral intravenous administration, intrathecal administration via external ventricular drainage and intracisternal administration via stereotactically placed catheters have been used and tested; yet, these alternative methods of administration remain to be of experimental character [16,17,18,19,20,21,22]. Furthermore, nimodipine has been administered intraarterially as a measure of local vasodilative rescue therapy in cases of symptomatic delayed cerebral vasospasm [17,23,24,25,26,27,28,29,30,31,32,33,34]. However, even though the intraarterial application of nimodipine has been available for more than a decade and seems to be safe, data on the effect of local intraarterial administration of nimodipine on functional outcome in patients with aSAH, who develop delayed vasospasm, remain very limited, and its effect on neurocognitive function has not been studied systematically at all [28,35]. Therefore, we examined the effect of local intraarterial catheter-based administration of nimodipine to treat delayed vasospasm after aSAH as part of the standard treatment protocol of our academic neurosurgical center on functional and neurocognitive outcome in patients with aSAH.

## 2. Materials and Methods

### 2.1. Patients

All patients aged 18 years and older with an angiographically proven, aneurysmal subarachnoid hemorrhage were eligible for inclusion in the study; patients with subarachnoid hemorrhage due to other causes or younger than 18 years were excluded. Prior to recruitment, all patients provided written informed consent in accordance with the Declaration of Helsinki; if their clinical condition hampered patients from providing written informed consent themselves, it was obtained by legal proxies. The study protocol was approved by the local standing committee on ethical practice of the University of Heidelberg (Approval Number S-642/2018). A total of 114 consecutive patients treated for aSAH of WFNS grades I–V at our institution between 1 May 2014 and 31 July 2020 were screened and met the inclusion criteria. As seven patients (6.5% of all screened patients) or their legal representatives did not provide written informed consent for study participation, a total of 107 patients were finally recruited.

### 2.2. Treatment Protocol

All patients were treated according to the local standard of care. The initial diagnosis of aSAH was obtained by computed tomography (CT) and CT angiography, as well as digital subtraction angiography (DSA). After interdisciplinary evaluation by the vascular neurosurgeon, interventional neuroradiologist and neurologist, the aneurysm was secured by either microsurgical clipping or endovascular coiling. If necessary, an external ventricular drainage was placed for CSF diversion, and if extubation was not feasible due to the patient’s clinical condition, an intraparenchymal probe for continuous ICP and ptiO_2_ monitoring was inserted. All patients were prophylactically treated with oral nimodipine (60 mg six times daily) to prevent DCI, and an euvolemic/normothermic regime was established. In addition to the continuous clinical monitoring, daily transcranial doppler sonography (TCD) was performed. DCI was defined as either one of the following: new focal neurological deficit or decrease in Glasgow Coma Score (GCS) of more than two points not attributable to other causes lasting for more than one hour, accelerated mean blood flow velocity in the middle cerebral artery (MCA) of more than 160 cm/s, impaired pTiO2 of less than 15 mmHg for more than one hour or perfusion deficit detected by perfusion CT [36]. If DCI was suspected, mean arterial blood pressure was raised stepwise and if symptoms did not resolve, a perfusion CT was performed. If a relevant perfusion deficit was present on CT scans, a DSA was performed.

Application of intra-arterial nimodipine was indicated at the discretion of the attending neurosurgeon and the interventional neuroradiologist in case of medically refractory angiographic cerebral vasospasm. Angiographic cerebral vasospasm was defined as narrowing of the arterial lumen in comparison to the initial angiography (on admission) as a reference and classified as ‘mild’ (<30% luminal narrowing), ‘moderate’ (30–50% luminal narrowing) or ‘severe’ (>50% luminal narrowing). Treatment was performed under conscious sedation or in general anesthesia via femoral arterial access. Intra-arterial nimodipine application was performed through a 5F diagnostic catheter placed either in the distal internal carotid artery (ICA) or the cervical vertebral artery (VA) depending on the location of angiographic cerebral vasospasm. In some patients, a superselective intra-arterial administration into the M1- or A1-segment of the medial cerebral artery/anterior cerebral artery (MCA/ACA) was performed via microcatheter. Up to 3 mg nimodipine per vessel were slowly infused over 30 min to avoid arterial hypotension; the typical dose of nimodipine, that was applied per single session, was 2 mg with doses ranging from 1 to 4 mg per application. Transient hypotensive effects were compensated by volume and/or vasopressor administration. After intra-arterial nimodipine treatment, a final angiography was performed and the angiographic treatment response was evaluated by two experienced interventional neuroradiologists by comparing the affected vessel diameter on the pre- and post-treatment angiogram. In cases of recurrent episodes of DCI and angiographically proven vasospasm, repetition of intraarterial treatment was possible.

### 2.3. Outcome Assessment

Patients were scheduled for follow-up visits 3-, 12- and 24-months after the hemorrhage. If their clinical condition hampered patients from following simple commands and, therefore, completing the neurological and neuropsychological evaluation, functional outcome was assessed using a structured GOSE telephone interview with the patient’s legal representatives. In addition, information gathered by the telephone interview were transferred to the modified Rankin Scale (mRS) to obtain an mRS score. Follow-up visits consisted of clinical examination conducted by an independent physician, assessment of GOSE and mRS as well as administration of the screening module of the Neurological Assessment Battery (NAB-S), which was conducted by a clinical neuropsychologist.

### 2.4. NAB-S

The Neuropsychological Assessment Battery screening module is part of the Neuropsychological Assessment Battery, which is a comprehensive, modular test battery, that was developed to evaluate neuropsychological function in neurologically impaired patients. It evaluates the five neuropsychological domains attention, language, memory, spatial und executive function and can be conducted within 45 min. After comparing the patient’s performance to age-, education- and gender-corrected norms and obtaining index values for each domain, results were stratified into six categories ranging from severely impaired to exceptional performance (severely impaired: index value more than two standard deviations below average; moderately impaired: index value between one and two standard deviations below average; mildly impaired: index value within one standard deviation below average; above average: within one standard deviation above average; exceptional: more than one standard deviation above average). While administration of the NAB-S to patients with aSAH is well feasible, we could recently show, that it can also effectively detect neuropsychological deficits relevant to the patients social and professional reintegration [37].

### 2.5. Statistical Analysis

Continuous variables were compared using one way ANOVA for normally distributed variables or Mann–Whitney-U-Test for nonnormally distributed variables. Discrete variables were compared using Chi^2^ or Fisher’s exact test, when appropriate. Statistical significance was defined as *p* ≤ 0.05.

## 3. Results

### 3.1. Baseline Characteristics

A total of 37 out of 107 patients developed DCI with angiographically proven flow restricting vasospasm; all of them subsequently received an intraarterial administration of nimodipine. In four patients, repetitive intraarterial nimodipine applications were performed due to recurrent flow restricting vasospasms (two patients with two and three applications, respectively). DCI was significantly less common in the conservative treatment group (100% vs. 32.9%, *p* < 0.001) and developed at 6.4 ± 3.6 and 6.3 ± 2.8 days after the bleeding, respectively. Patients in the intervention group were affected more severely, as significantly more patients suffered from high-grade aSAH (WFNS IV and V) compared to the conservative treatment group (54.1% vs. 31.4%, *p* = 0.04) and required initial placement of an external ventricular drainage (EVD) for the treatment of acute hydrocephalus significantly more often (97.3% vs. 80.0%, *p* = 0.03). In addition, duration of ICU treatment was significantly longer in patients, who had received interventional therapy (23.9 ± 8.7 vs. 19.8 ± 10.7 days, *p* = 0.02). Mean age as well as aneurysm size did not differ between the two groups (57.1 ± 11.2 vs. 56.9 ± 13.0 years and 7.1 ± 3.9 vs. 6.9 ± 3.7 mm) and in both cohorts, the majority of aneurysms were located in the anterior circulation (90.2% vs. 90.1%). Treatment modalities were distributed evenly between clipping and coiling in both groups (48.8% for both in the intervention group and 54.9% and 43.7% in the conservative treatment group, respectively) and there was no difference in the frequency of decompressive hemicraniectomy and in-hospital mortality (27.0% vs. 15.7% and 16.2% vs. 11.4%, respectively). Baseline characteristics are shown in Table 1.

### 3.2. Functional Outcome

Even though patients in the intervention group were affected more severely upon admission to the ICU, significant differences in functional outcome between the intervention group and the control were only present at discharge as well as after three months (favorable outcome defined as a GOSE of more than four points or an mRS of less than three points: 8.1% vs. 41.4%, *p* < 0.001 and 10.8% vs. 47.1%, *p* < 0.001, respectively, at discharge; 28.6% vs. 72.7%, *p* = 0.01 and 42.9% vs. 84.8%, *p* = 0.01, respectively, at three months), while this difference had levelled off by 12-months after the bleeding and was not present at 24-months as well (41.2% vs. 69.2% and 52.9% vs. 76.9%, respectively, at twelve months; 50.0% vs. 82.4% and 70.0% vs. 70.6%, respectively, at 24-months). Data on functional outcome are summarized in Table 2.

### 3.3. Neuropsychological Outcome

In the interventional group, the NAB-S could not be conducted due to the patient‘s clinical condition in one patient at three and in two patients at twelve months, while this was the case for eight and two patients in the conservative group, respectively.

As with functional outcome, despite initially being affected more severely, severe impairment of any of the five neuropsychological domains attention, language, memory, spatial and executive function was present significantly more often in patients treated interventionally compared to conservatively treated patients only at three and twelve months (76.9% vs. 36.0%, *p* = 0.04 at three months, and 66.7% vs. 29.2%, *p* = 0.05 at twelve months), while this difference was not statistically significant at 24-months (50.0% vs. 23.5%) any more. There was no statistically significant difference in the presence of moderate impairment of one or more domains between the groups at any timepoint (76.9% vs. 48.0% at three months; 66.7% vs. 54.2% at twelve months; 80.0% vs. 47.1% at 24-months).

By three months after the bleeding, only 10.3% of the patients in the conservative treatment group and no patient in the intervention group had been able to return to work. While patients treated interventionally were significantly less likely to return to work at twelve months after the bleeding (5.9% vs. 38.1%, *p* = 0.03), there was no significant difference between the two groups at 24-months after the hemorrhage (22.2% vs. 46.7%). Table 3 summarizes the neuropsychological outcome.

### 3.4. Complications and Side Effects

There were no accidental vessel perforations, intolerable drops of blood pressure or pulmonary insufficiencies due to pulmonary shunting during interventional procedures. Furthermore, there were no puncture site complications requiring special treatment and no cases of discontinuation of oral nimodipine therapy due to delayed complications of intraarterial application of nimodipine (e.g., persisting arterial hypotension or pulmonary insufficiency). Overall, there were more pulmonary complications during ICU treatment in the interventional group (54.1% vs. 35.7%), which was mainly due to a higher rate of pneumonia (45.9% vs. 27.1%); however, these differences were not statistically significant and none of the pulmonary complications could be directly linked to the intraarterial application of nimodipine (most pneumonias were due to aspiration and already present at ICU admission). Pulmonary embolism (two cases), respiratory insufficiencies not related to infection (three cases) and neurogenic pulmonary edema (one case) were only observed in the conservative treatment group. Finally, the rate of ventriculitis, which was defined as a rise in body temperature >38.5 °C for more than two hours or elevated blood leukocyte count or procalcitonin levels in conjunction with an elevated cell count in the CSF as well as verification of bacterial CSF infection by Gram staining or PCR analysis, did not differ between the two groups (10.8% vs. 12.9%, respectively).

## 4. Discussion

Intraarterial rescue therapy with nimodipine might positively affect long-term functional outcome.

In the current study, we prospectively evaluated short- and long-term functional outcome by means of mRS and GOSE for up to 24-months after aSAH. Even though intraarterial catheter-based rescue therapy for delayed vasospasm following aSAH has been available for more than a decade, very few studies have reported the effect of this treatment option on functional outcome, as most studies have focused on safety and short-term efficacy [17,23,24,28,35]. Two notable studies reporting on functional outcome in a prospectively recruited cohort are those by Hänggi et al. and Weiss et al. published in 2008 and 2019, respectively [17,28]. Favorable outcomes were reported in 61% and 42% of the patients, who had received intraarterial rescue therapy, at three months, respectively, while in our study, favorable outcome was achieved in 43% (mRS three or less) and 29% (GOSE five or more) of the patients in the interventional treatment group [17,28]. However, in the studies by Hänggi et al. and Weiss et al., significantly less patients with poor grade aSAH compared to the interventional treatment group of our study (31% and 18% versus 54%) were included in the analyses. As WFNS grade significantly correlates with the outcome after aSAH, short-term functional outcome in our study seems to be comparable to the reported outcome in previous studies. However, no data on long-term effects of intraarterial rescue therapy with nimodipine has been available so far; therefore, we provide information on this issue for the first time.

In our study, significantly more patients suffered from high-grade aSAH and developed DCI in the interventional treatment group compared to the conservative treatment group. Furthermore, significantly more patients in the intervention group required continuous CSF drainage at discharge. Therefore, as higher WFNS grades, the development of DCI and the presence of hydrocephalus are predictors for poor outcome after aSAH, one would expect more unfavorable functional outcomes in the interventional treatment group [1,38]. However, in the current study, this was only the case at discharge and three months after the bleeding. At 12- and 24-months after the hemorrhage, no significant differences in favorable outcome between the two groups could be detected, suggesting a potential modest favorable effect of the intraarterial rescue therapy with nimodipine. This is especially important to mention, as previous studies, that have only assessed short-term outcome, might have underestimated this potential delayed treatment effect.

Finally, in the current study, outcome data of patients treated with intraarterial rescue therapy was compared to a cohort of patients treated at the same neurointensive care unit using the same treatment protocol at the same time, while previous studies lacked control groups and only compared outcome data with historical cohorts, which might have been treated in different clinical environments and according to different treatment algorithms [17,28]. However, as a limiting factor, it has to be mentioned, that in our study, outcomes between two uncontrolled cohorts of patients were compared; therefore, rather than proving a distinct causative relation between the intraarterial nimodipine treatment and improved patient outcomes, we provide prospectively obtained data on the clinical course of patients receiving intraarterial rescue therapy and those without the need for rescue treatment.

### 4.1. Intraarterial Rescue Therapy with Nimodipine Might Potentially Improve Neurocognitive Outcome

Recovery of neurocognitive function has been recognized as one of the most important factors of successful recovery as well as social and professional reintegration after suffering from aSAH; however, no study has systematically evaluated the effects of the intraarterial rescue therapy for delayed vasospasm after aSAH on neuropsychological outcome so far [39,40,41,42]. Even though significant impairment of neurocognitive function was present in both groups throughout the follow-up period and only few patients managed to return to work to their previous capacity, a temporal pattern of recovery similar to the one seen in functional outcome could be observed: Severe neurocognitive deficits were detected in significantly more patients, that needed intraarterial rescue therapy, compared to conservatively treated patients at short-term follow-ups, while this difference had diminished after 24-months following bleeding. When only assessing moderate neurocognitive dysfunction, there was no significant difference between the two treatment groups at any timepoint, indicating a potential moderate positive effect of the intraarterial rescue therapy on neuropsychological outcome.

Neuropsychological function is closely linked to regaining the ability to work at a pre-injury capacity. Therefore, it comes as no surprise, that a similar temporal pattern of recovery could be detected, when assessing the ability to return to work as well: While significantly less patients in the intervention group had returned to work at twelve months, no significant difference between the two groups could be detected at long-term follow-up [37,43].

### 4.2. Comparison of (Repeated) Single Dose Intraarterial Nimodipine versus Continuous Intraarterial Application

In our study, all patients in the intervention group were treated with local single dose intraarterial nimodipine, that could be repeated, if severe vasospams recurred; however, continuous intraarterial administration of nimodipine has also been proposed as an effective treatment option for delayed vasospasm. Bele et al. described a potential beneficial effect of continuous intraarterial application of nimodipine as a rescue measure for patients with aSAH, who developed clinically relevant vasospasms refractory to conservative measures [29]. However, in this study, the interpretation of a potential treatment effect was based on the comparison to historical cohorts, that had been treated at different intensive care units with different conservative treatment protocols. Bele et al. reported, that in their study, 48% of the patients suffered from high-grade aSAH and 76% of the patients reached a favorable functional outcome assessed by GOS at six months [29]. Even though the proportion of high-grade aSAH patients was similar to our interventional treatment group, functional outcome was less favorable in our study throughout the entire follow-up period; yet, the outcome described by Bele et al. seems to be very favorable compared to previously reported outcomes after aSAH [44,45]. Furthermore, the effect of the continuous administration of nimodipine on long-term outcome remains unclear and although the continuous intraarterial application of nimodipine seems to have beneficial effects on short-term neurological function as well as angiographic vasospasm, the rate of complications appear to be high and management of these complications is complex [33]. In contrast, in our study, there were no acute procedure-related complications and even though overall pulmonary complications during the course of ICU treatment were more frequently observed in the interventional group, these complications were manageable and could not be directly linked to the intraarterial nimodipine administration. Furthermore, as significantly more patients in the intervention group suffered from high-grade aSAH compared to the conservative group, those patients were on mechanical ventilation more frequently and for a longer period of time; therefore, as mechanical ventilation represents one of the most important risk factors for pulmonary complications such as infection, the higher number of pulmonary complications in the intervention group was expected and cannot be related to the more invasive vasospasm treatment.

Finally, the comparison between the two treatment modalities of continuous versus single dose administration of nimodipine is further aggravated by different inclusion criteria and treatment algorithms; therefore, choosing the best algorithm of the intraarterial administration of nimodipine remains difficult and prospective studies directly comparing both treatment regimens and including a control group should be undertaken to answer this question.

## 5. Limitations

In the current study, patients who received an invasive treatment were compared to patients without the need for interventional treatment for symptomatic vasospasm. As allocation to either cohort was not randomized, but rather based on the clinical need for interventional treatment according to the standard treatment protocol instead, the conservative treatment group was not a true control group in our study; however, even though it is scientifically required to rigorously evaluate the effect of the interventional rescue therapy, respecting the declaration of Helsinki and thoroughly considering ethical aspects, including a control group depriving patients with refractory symptomatic vasospasm of a potentially beneficial rescue therapy would be ethically difficult to justify.

In addition, we report the results of our single-center experience. On the one hand, this limits generalizability of the results, as they were obtained in the specific environment of our ICU and based on our own treatment protocol. On the other hand, patient numbers are limited, which especially affects the interpretation of long-term follow-up data. Therefore, conducting a multicenter-trial to increase patient numbers and to improve the validity of data analysis would be desirable, especially with the aim of long-term follow-up data collection, which was somewhat limited in our study, as a certain number of patients were not available for analysis at long-term follow-ups for various reasons.

Furthermore, we cannot report any information on patient’s premorbid status due to the acute nature of aSAH. Therefore, differentiating between neuropsychological impairments, which had already existed before the hemorrhage, and impairments that were caused by the acute event, is difficult. However, as this is true for both the interventional and the conservative cohort, evaluation of the effect of the rescue treatment is still possible.

Finally, as aSAH is a very complex disease with a plethora of parameters that might influence the clinical and neuropsychological outcome, many of which remain understudied, assessing possible effects of one single intervention in a neurointensive care setting is very difficult. Even though we assessed parameters, that are known to influence clinical outcome in aSAH patients, such as the WFNS grade, the development of DCI, ventriculitis and pulmonary complications as well as procedure-related complications, our study results need to be interpreted cautiously, as other important factors that might have influenced the observed outcome, might have been missed.

## 6. Conclusions

Even though patients, who received intraarterial nimodipine as a rescue measure to treat angiographically proven, symptomatic, delayed vasospasm, were initially significantly more severely impaired, there were no significant differences in functional and neuropsychological outcomes at medium- and long-term follow-up between interventionally and conservatively treated patients. Therefore, our data suggest that the local intraarterial administration of nimodipine as a rescue treatment for vasospasm after aSAH potentially might have a beneficial effect on functional as well as on neuropsychological outcomes and professional reintegration. However, prospective, randomized, and controlled studies are needed to precisely assess the actual treatment effect of the intraarterial nimodipine application to address delayed symptomatic vasospasm after aSAH and to determine the most effective method of nimodipine administration.

## Figures and Tables

**Table 1:** Baseline characteristics. WFNS: World Federation of Neurological Surgeons; EVD: External ventricular drainage; DCI: Delayed cerebral ischemia; ICU: Intensive care unit; CSF: Cerebrospinal fluid.

	Interventional Group	Conservative Group	*p*-Value
Number	34.6% (37/107)	65.4% (70/107)	
Age (y)	57.1 ± 11.2	56.9 ± 13.0	0.96
Male	24.3% (9/37)	51.4% (36/70)	**0.01**
WFNS I-III	45.9% (17/37)	68.6% (48/70)	**0.04**
WFNS IV + V	54.1% (20/37)	31.4% (22/70)	**0.04**
Fisher III + IV	100% (37/37)	88.1% (59/67)	0.07
Ant. Circulation *	90.2% (37/41)	90.1% (64/71)	0.76
Aneurysm size (mm)	7.1 ± 3.9	6.9 ± 3.7	0.76
Clipping **	48.8% (20/41)	43.7% (31/71)	0.74
Coiling **	48.8% (20/41)	54.9% (39/71)	0.74
Initial EVD	97.3% (36/37)	80.0% (56/70)	0.03
Decompression	27.0% (10/37)	15.7% (11/70)	0.25
DCI	100% (37/37)	32.9% (23/70)	**<0.001**
Days to DCI	6.4 ± 3.6	6.3 ± 2.8	0.95
Ventriculitis	10.8% (4/37)	12.9% (9/70)	1.0
ICU stay (d)	23.9 ± 8.7	19.8 ± 10.7	**0.02**
In-Hospital-Mortality	16.2% (6/37)	11.4% (8/70)	0.69
CSF Drainage at Discharge ***	67.7% (21/31)	38.7% (24/62)	**0.02**

* In two cases in the intervention group (two aneurysms in one and three aneurysms in another case) and in one case in the conservative group (two aneurysms), more than one aneurysm was treated within the initial ICU stay. ** In one case in the intervention group aneurysm treatment was postponed due to vasospasm on initial angiogram and in one case in the conservative group therapy was limited was due to poor clinical prognosis. *** As mortality was 6 of 37 and 8 of 70 patients in the respective groups, the necessity for CSF drainage at discharge was assessed in 31 and 62 patients, respectively.

**Table 2:** Functional outcome assessed by GOSE and mRS. GOSE: Extended Glasgow Outcome Scale; mRS: modified Rankin Scale. A GOSE score of five or better indicates independence at home but inability to return to work. An mRS score of three or less indicates the ability to walk without assistance but requiring some help with daily activities.

		Interventional Group	Conservative Group	*p*-Value
Discharge	GOSE > 4	8.1% (3/37)	41.4% (29/70)	**<0.001**
	mRS < 4	10.8% (4/37)	47.1% (33/70)	**<0.001**
3 Months	GOSE > 4	28.6% (4/14)	72.7% (24/33)	**0.01**
	mRS < 4	42.9% (6/14)	84.8% (28/33)	**0.01**
12 Months	GOSE > 4	41.2% (7/17)	69.2% (18/26)	0.13
	mRS < 4	52.9% (9/17)	76.9% (20/26)	0.19
24 Months	GOSE > 4	50.0% (5/10)	82.4% (14/17)	0.10
	mRS < 4	70.0% (7/10)	70.6% (12/17)	1.0

**Table 3:** Neuropsychological outcome assessed by NAB-S. NAB-S: Screening module of the Neuropsychological Assessment Battery.

		Interventional Group	Conservative Group	*p*-Value
NAB-S severely impaired	3 Months	76.9% (10/13)	36.0% (9/25)	**0.04**
	12 Months	66.7% (10/15)	29.2% (7/24)	**0.05**
	24 Months	50.0% (5/10)	23.5% (4/17)	0.22
NAB-S moderately impaired	3 Months	76.9% (10/13)	48.0% (12/25)	0.17
	12 Months	66.7% (10/15)	54.2% (13/24)	0.66
	24 Months	80.0% (8/10)	47.1% (8/17)	0.12
Ability to work	3 Months	0% (0/13)	10.3% (3/29)	0.54
	12 Months	5.9% (1/17)	38.1% (8/21)	**0.03**
	24 Months	22.2% (2/9)	46.7% (7/15)	0.39

## Data Availability

The datasets used and/or analyzed during the current study are available from the corresponding author on reasonable request.

## Abbreviations

aSAH	aneurysmal subarachnoid hemorrhage
CT	computed tomography
DCI	delayed cerebral ischemia
DSA	digital subtraction angiography
EVD	external ventricular drainage
F	French
GCS	Glasgow Coma Score
GOSE	Extended Glasgow Outcome Scale
ICA	Internal carotid artery
ICP	intracranial pressure
ICU	intensive care unit
MCA	middle cerebral artery
mRS	modified Rankin Scale
NAB-S	Neurological Assessment Battery screening module
pTiO2	oxygen partial pressure
TCD	transcranial doppler sonography
VA	Vertebral artery
